# Recurrence of cervical intraepithelial neoplasia grades 2 or 3 in HIV-infected women treated by large loop excision of the transformation zone (LLETZ)

**DOI:** 10.1590/S1516-31802008000100004

**Published:** 2008-01-03

**Authors:** Fábio Russomano, Aldo Reis, Maria José Camargo, Beatriz Grinsztejn, Maria Aparecida Tristão

**Keywords:** Cervical intraepithelial neoplasia, Prognosis, HIV seropositivity, Recurrence, Electrosurgery, Neoplasia intra-epitelial cervical, Prognóstico, Soropositividade para HIV, Recidiva, Eletrocirurgia

## Abstract

**CONTEXT AND OBJECTIVE:**

Women infected by HIV are more likely to have cervical cancer and its precursors. Treatment of the precursor lesions can prevent this neoplasia. The aim of this study was to assess the likelihood of recurrent cervical intraepithelial neoplasia grades 2 or 3 (CIN 2-3) in HIV-infected women, compared with HIV-negative women, all treated by large loop excision of the transformation zone (LLETZ).

**DESIGN AND SETTING:**

A cohort study in Instituto Fernandes Figueira, Fundação Oswaldo Cruz (IFF-Fiocruz), Rio de Janeiro.

**METHOD:**

55 HIV-positive and 212 HIV-negative women were followed up after LLETZ for CIN 2-3 (range: 6-133 months).

**RESULTS:**

The incidence of recurrent CIN 2-3 was 30.06/10,000 woman-months in the HIV-positive group and 4.88/10,000 woman-months in the HIV-negative group (relative risk, RR = 6.16; 95% confidence interval, CI: 2.07-18.34). The likelihood of recurrence reached 26% at the 62^nd^ month of follow-up among the HIV-positive women, and remained stable at almost 0.6% at the 93^rd^ month of follow-up among the HIV-negative women. We were unable to demonstrate other prognostic factors relating to CIN recurrence, but the use of highly active antiretroviral therapy (HAART) may decrease the risk of this occurrence among HIV patients.

**CONCLUSION:**

After LLETZ there is a higher risk of recurrence of CIN 2-3 among HIV-positive women than among HIV-negative women. This higher risk was not influenced by margin status or grade of cervical disease treated. The use of HAART may decrease the risk of this occurrence in HIV patients.

## RESUMO

**CONTEXTO E OBJETIVO:**

Mulheres infectadas pelo HIV têm maior probabilidade de apresentar câncer cervical e seus precursores. O tratamento dessas lesões pode prevenir a neoplasia. O objetivo deste estudo foi verificar a probabilidade de recorrência de neoplasia intra-epitelial cervical graus 2 ou 3 (NIC 2-3) em mulheres infectadas pelo HIV (HIV+), comparando-a com a de mulheres soronegativas (HIV-) tratadas pela exérese da zona de transformação por alça diatérmica (EZTAD).

**TIPO DE ESTUDO E LOCAL:**

Estudo de coorte conduzido no Instituto Fernandes Figueira — Fundação Oswaldo Cruz (IFF-Fiocruz), Rio de Janeiro, Brazil.

**MÉTODO:**

55 HIV+ e 212 HIV- foram acompanhadas após tratamento de NIC 2-3 pela EZTAD (faixa: 6-133 meses).

**RESULTADOS:**

A incidência de NIC 2-3 recorrente foi de 30,06/10.000 mulheres-mês no grupo HIV+ e 4,88/10.000 mulheres-mês no grupo HIV- (risco relativo, RR = 6,16; intervalo de confiança, IC 95%: 2,07-18,34). A probabilidade de recorrência alcançou 26% aos 62 meses de acompanhamento em mulheres HIV+, e manteve-se estável em cerca de 0,6% no 93° mês de acompanhamento em mulheres HIV-. Não pudemos demonstrar outros fatores prognósticos relacionados à recorrência de NIC, mas o uso de terapia antiretroviral potente (highly active antiretroviral therapy - HAART) pode reduzir o risco dessa ocorrência em pacientes HIV+.

**CONCLUSÕES:**

Mulheres HIV+ têm maior risco de recorrência de NIC 2-3 após EZTAD comparadas a mulheres HIV-. Esse maior risco não foi influenciado pelo status da margem ou grau de doença tratada. O uso de HAART pode reduzir o risco desta ocorrência em mulheres HIV+.

## Introduction

The HIV epidemic is worldwide. The Joint United Nations Programme on HIV/AIDS (UNAIDS) has indicated that more than 39 million people were living with HIV around the world at the end of 2006, of which 1.7 million were in Latin America and one third of these in Brazil.^1^

The improving survival rates observed in Brazil and other countries are a consequence of better clinical management, prophylaxis against common infections and the use of highly active antiretroviral therapy (HAART).^1–3^ These factors have turned attention towards chronic and degenerative diseases that were, until now, irrelevant. For example, cervical precancer is more prevalent among HIV-positive patients,^4–8^ and is more likely to progress to a higher grade of the disease.^9,10^

Several studies have consistently shown that HIV-positive women present higher risk of cervical intraepithelial neoplasia (CIN) persistence or recurrence after standard therapy.^9,11–16^ These features have led some clinicians to reevaluate the efficacy of traditional therapy for CIN grades 2 or 3 (CIN 2-3) among HIV-infected women.^17–22^ However, prognostic factors such as CD4 count, positive margins from excisional procedures and use of HAART have not been consistently correlated with this event.^12,15,16,23–25^

## OBJECTIVE

The aim of this study was to report on the incidence of recurrent disease after large loop excision of the transformation zone (LLETZ)^26^ performed to treat CIN 2-3 in HIV-infected women in Rio de Janeiro, Brazil; and on their relative risk compared with HIV-negative women and the likelihood of this occurrence over time.

## Materials and methods

Fifty-five HIV-infected and 212 HIV-negative women were followed up after treatment for CIN 2-3 in Rio de Janeiro, Brazil, from 1994 to 2006. All these patients underwent LLETZ at Instituto Fernandes Figueira, Fundação Oswaldo Cruz (IFF-Fiocruz). All the cases presented satisfactory colposcopy examinations before treatment, and the transformation zones were fully visible in the ectocervical region or within the first centimeter of the endocervical canal.

LLETZ was performed under local anesthesia in an outpatient setting. The histological specimen was comprehensively examined in order to rule out invasion. Two tests, enzyme-linked immunosorbent assay (ELISA) and immunofluorescence, were performed on two different samples to detect any cases of HIV seropositivity. HIV absence was defined as a negative ELISA test at the time of patient inclusion and two and four years after treatment.

During the follow-up, all of the patients underwent a Pap smear examination and, on a subsequent visit, colposcopy was performed by one of the investigators (FR or MJC), every six months. Patients who failed to show up for any appointment received a letter or personal contact to schedule their next medical visit.

When an atypical area was observed, a new biopsy was taken for histological examination. Recurrence was documented when CIN 2-3 or worse was reported.

Information about possible confounding factors was collected from the histological reports on the LLETZ specimen. These were the presence of disease at the surgical margins and the grade of CIN treated (CIN 2 or CIN 3). Among HIV patients, we considered the CD4 cells count per mm^3^ and the use of HAART to be possible prognostic factors.

Although all our HIV-infected patients were attended by infectious disease specialists within public hospital settings where CD4 count testing was available and antiretroviral therapy was available free of charge, we had no consistent information regarding CD4 count results, or about who was using this testing method, or whether all of our HIV patients had adhered to this. Because of this limitation, we took into consideration CD4 counts carried out less than 90 days before or after the last appointment (or the date of detecting recurrence) and the use of HAART at this time, only for those patients for whom this information was accurate.

In order to calculate the sample size, we used an estimate of seven times greater risk (which was our previously observed relative risk between these two groups), alpha error of 5%, power of 80%, the ratio of non-HIV-infected women to HIV-infected women in our setting (4:1) and an expected incidence of recurrent CIN 2-3 in unexposed subjects of 2.6%. This gave us a sample size of 245 women (196 HIV-negative and 49 HIV-infected women) (using Epi-Info version 6.04d).

The information on each visit was entered into a database and the analyses were performed using the Statistical Package for the Social Sciences (SPSS) software (version 8.0 – SPSS Inc, 1997) and Epi-Info (version 6.04d).

The characteristics of the study population are shown in Table 1.

**Table 1.** Study population (Rio de Janeiro, Brazil, 2006)

Characteristics	HIV-positive	HIV-negative	p-value
n (%)	55 (20.6)	212 (79.4)	-
**Age (years)**			
Mean (SD)	31.56 (7.19)	32.36 (7.61)	0.468*
Median (variance)	31.35 (51.642)	31.83 (57.922)	
Minimum-maximum	19-51	17-54	
**Age at end of follow-up period (years)**			
Mean (SD)	35.09 (7.41)	37.20 (8.31)	0.070*
Median (variance)	34.23 (54.931)	37.01 (69.118)	
Minimum-maximum	23-54	19-63	
**Length of follow-up after LLETZ (months)**			
Mean (SD)	42.35 (27.8)	58.04 (31.09)	0.000*
Median (variance)	33.40 (773.068)	60.53 (966.409)	
Minimum-maximum	7-113	6-133	
**CIN grade treated**			
CIN-2 (% in row - % in column)	32 (28.6 - 58.2)	80 (71.4-37.7)	0.02†
CIN-3 (% in row - % in column)	20 (13.2-36.4)	131 (86.8-61.8)	
CIN 2-3‡ (% in row - % in column)	3 (75-5.5)	1 (25-0.5)	
**Margin involvement in LLETZ specimen**			
Endocervical margin (% in row - % in column)	13 (28.9-23.6)	32 (71.1-15.1)	0.112†
Endocervical margin not accessed or damaged§ (% in row - % in column)	2 (40.0-3.6)	3 (60.0-1.4)	
Ectocervical margin (% in row - % in column)	12 (35.3-21.8)	22 (64.7-10.4)	0.02†
Ectocervical margin not accessed or damaged§ (% in row - % in column)	2 (33.3-3.6)	4 (66.7-1.9)	
Stromal margin (% in row - % in column)	1 (100-1.8)	0	0.205||
Stromal margin not accessed or damaged§ (% in row - % in column)	1 (25.0-1.8)	3 (75.0-1.4)	
At least one margin involved (% in row - % in column)§	20 (31.3-36.4)	44 (68.8-20.7)	0.016†
Mean CD4 cell/mm^3^ count (SD)¶	557.42 (295.59)	-	-
% using HAART**	84.6	-	-

SD = standard deviation; LLETZ = large loop excision of the transformation zone; CIN = cervical intraepithelial neoplasia; HAART = highly active antiretroviral therapy.

* Two-tailed Student's t test, without assuming equal variance;

† Chi-squared test;

‡ In these cases it was not possible to differentiate CIN-2 from CIN-3 (excluded from chi-squared statistics);

§ Including cases in which margin assessment was impossible due to thermal artifact or specimen segmentation (these cases were excluded from the hypothesis test of association with recurrence of disease);

|| Fisher's exact test;

¶ For 19 HIV patients for whom this count was available 90 days before or after the last appointment;

** For 26 HIV patients for whom this information was available at the last appointment.

In order to accommodate different follow-up periods, we used the person-length-of-observation concept to give us numbers for estimating absolute and relative risks of recurrence. To estimate the risk of recurrence over the course of the follow-up, we performed survival analysis using the Kaplan-Meyer method (SPSS, version 8.0).

The local Ethics Committee approved the study protocol and all patients signed an informed consent statement before inclusion.

## Results

We found seven cases of recurrence in the HIV-positive group and six in the control group. This produced an incidence of recurrence of CIN 2-3 of 30.06/10,000 woman-months in the HIV-infected group and 4.88/10,000 woman-months in the HIV-negative group. This resulted in a relative risk (RR) of 6.16 (95% confidence interval, CI: 2.07-18.34) (Table 2).

**Table 2.** Incidence and recurrence of risk of cervical intraepithelial neoplasia (CIN 2-3) in study groups (Rio de Janeiro, Brazil, 2006)

	HIV-positive	HIV-negative
Number of patients	55	212
Number of recurrences	7	6
Total number of months of follow-up	2,329	12,305
Incidence (in woman-months)	30.06/10,000	4.88/10,000
Incidence (in woman-years)	3.61/100	0.58/100
Overall incidence (in woman-months)	8.88/10,000	
Overall incidence (in woman-years)	1.07/100	
Relative risk (95% CI)	6.16 (2.07-18.34)	

CI = confidence interval.

**Figure 1:**
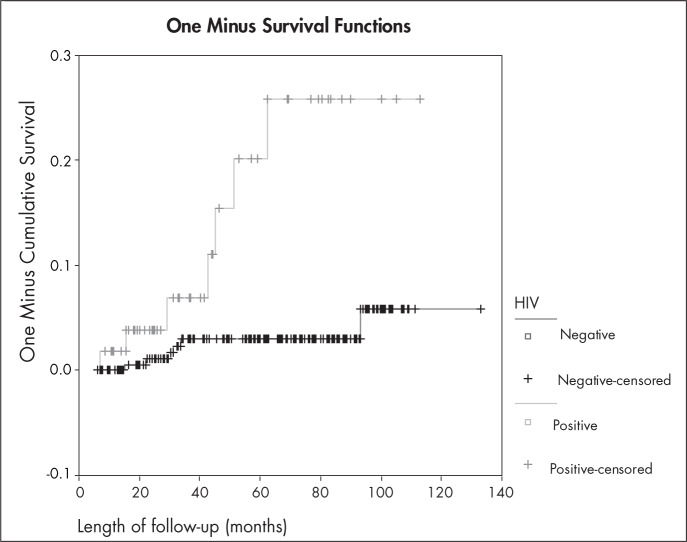
Likelihood of recurrence during follow-up period, for the two study groups, obtained using the Kaplan-Meyer method (1-survival function). Log-rank test = 14.32; p = 0.0002 (Rio de Janeiro, Brazil, 2006).

The risk of recurrence over time is shown in Figure 1, as obtained using the Kaplan-Meyer method (1-survival). The difference between the curves showed statistical significance (log-rank test = 14.32; p = 0.0002).

Table 3 shows the relationships between possible prognostic factors for recurrence of CIN, in order to highlight any confounding factors.^27^ CIN grade and margin involvement failed to show any significant relationship with the outcome. Length of follow-up was not equally distributed between the groups, in relation to the outcome.

**Table 3.** Possible prognostic factors for cervical intraepithelial neoplasia (CIN 2-3) recurrence (Rio de Janeiro, Brazil, 2006)

	Recurrence detected	Recurrence not detected	p-value
**Cases (%)**	13 (4.9)	254 (95.1)	
**Age at time of performing LLETZ – mean (SD)**	32.5 (7.2)	32.2 (7.6)	0.880*
**Age at end of follow-up period – mean (SD)**	35.6 (7.5)	36.9 (8.2)	0.563*
**Length of follow-up in months – mean (SD)**	36.8 (22.9)	55.7 (31.2)	0.013*
**CIN grade treated – n (%)**			
CIN 2 (% in row; % in column)	7 (6.3-53.8)	105 (93.8-41.3)	0.40†
CIN 3 (% in row; % in column)	6 (4.0-46.2)	145 (96.0-57.1)	
CIN 2-3‡ (% in row; % in column)		4 (100.0-1.6)	
**LLETZ specimen margin involvement**			
Any involvement (% in row; % in column)	2 (3.1-15.4)	62 (96.9-24.4)	
No involvement§ (% in row; % in column)	11 (5.4-84.6)	192 (94.6-75.6)	0.74||

SD = standard deviation; LLETZ = large loop excision of the transformation zone; CIN = cervical intraepithelial neoplasia.

* Student's t test, without assuming equal variance;

† Chi-squared test;

‡ Not possible to differentiate between CIN-2 or 3 and therefore excluded from the statistical test of association of CIN grade with recurrence;

§ Including cases in which margin involvement could not be assessed due to thermal artifact or excessive fragmentation;

|| Fisher's exact test.

**Figure 2:**
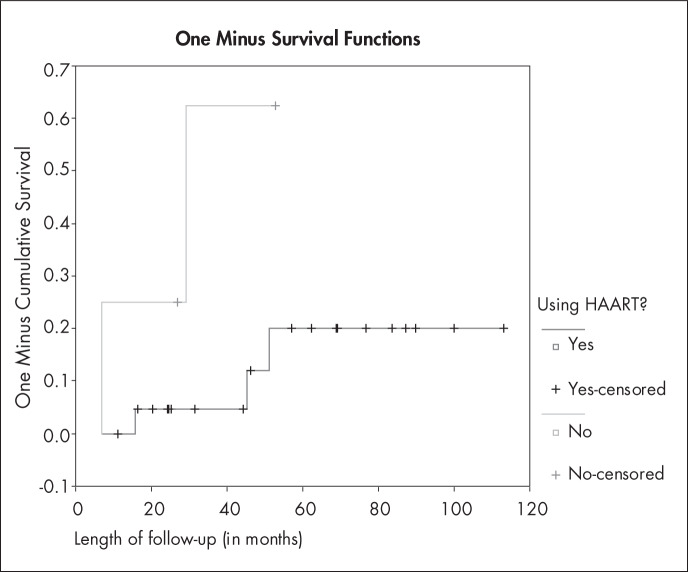
Likelihood of recurrence during follow-up period for 26 HIV patients, according to whether they were using highly active antiretroviral therapy (HAART), using the Kaplan-Meyer method (1-survival function). Log-rank test = 4.32; p = 0.0377 (Rio de Janeiro, Brazil, 2006).

**Figure 3:**
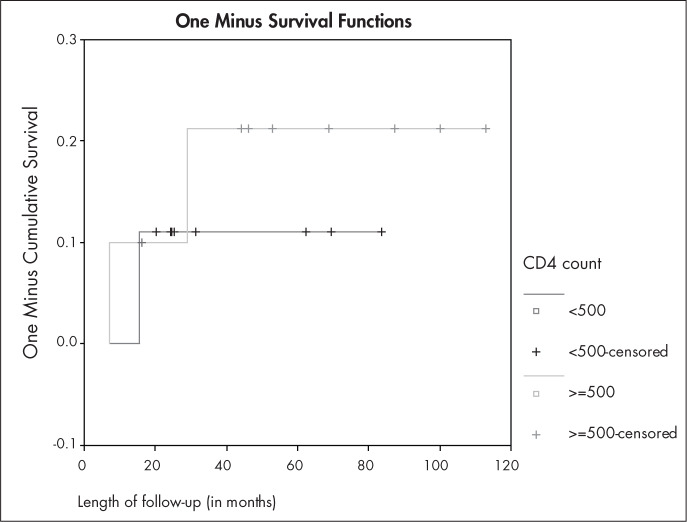
Likelihood of recurrence during follow-up period for 19 HIV patients, according to CD4 count (less than 500 cells/mm3 versus 500 cells/mm3 or more), using the Kaplan-Meyer method (1-survival function). Log-rank test = 0.13; p = 0.7178 (Rio de Janeiro, Brazil, 2006).

Analysis of CD4 count and HAART use also failed to demonstrate any statistically significant relationship, which was probably due to the small number of patients from whom this information was available (Table 4). However, HIV patients using HAART seemed to have less risk of recurrence than did HIV patients who were not using it. This is shown in Figure 2, in which the Kaplan-Meyer method was used to compare the likelihood of recurrence between these two groups (log-rank test = 4.32; p = 0.0377). This trend can also be seen in Figure 3, which shows that the recurrence of CIN 2-3 was more frequent in HIV patients who had CD4 counts of less than 500 cells/mm^3^, but the difference between these two curves was not significant (Log-rank test = 0.13; p = 0.7178).

**Table 4.** Possible prognostic factors for cervical intraepithelial neoplasia (CIN 2-3) recurrence in HIV patients (Rio de Janeiro, Brazil, 2006)

	Recurrence detected	Recurrence not detected	p-value
Cases (%)	7 (12.7)	48 (87.3)	
Less than 500 CD4 cells/mm^3^ (% in row - % in column)*	2 (20.0-66.7)	8 (80.0-50.0)	
500 CD4 cells/mm^3^ or more (% in row - % in column)*	1 (11.1-33.3)	8 (88.9-50.0)	1.00†
Not using HAART‡	2 (50.0-40.0)	2 (50.0-9.5)	
Using HAART‡	3 (13.6-60.0)	19 (86.4-90.5)	0.155†

HAART = highly active antiretroviral therapy.

* For 19 HIV patients for whom this count was available 90 days before or after the last appointment;

† Fisher's exact test;

‡ For 26 HIV patients for whom this information was available at the last appointment.

Patients who had not attended any visit over the last year of the study, or had asked to leave the study, or had left the cohort for other reasons, were considered to have been lost from the follow-up. Table 5 shows the numbers of losses in each group and the known reasons for this event.

**Table 5.** Losses in each study group and the known reasons for this event (Rio de Janeiro, Brazil, 2006)

	HIV-positive	HIV-negative	Total
**Number of patients (% within group)**	**16 (29.1)**	**103 (41.4)**	**119 (44.6)**
Asked to leave the study (% of lost patients)	3 (18.8)	30 (29.1)	33 (27.7)
Had hysterectomy (% of lost patients)	1 (6.3)		1 (0.8)
Died due to causes unrelated to cervical cancer (% of lost patients)	1 (6.3)		1 (0.8)
Did not show up during last year of follow up, for unknown reasons (% of lost patients)	11 (68.8)	73 (70.9)	84 (70.6)

Reanalyzing the censored cases, if it were considered that all of the lost HIV-positive patients had presented recurrence and none of the lost HIV-negative patients had had recurrence of CIN 2-3, the incidence of this outcome in each group would have been 987.5/10,000 patient-months and 48.0/10,000 patient-months, respectively, and the RR would have been as high as 20.56 (95% CI = 8.37-50.50). Similarly, if it were considered that none of the lost HIV-positive patients had presented this outcome and all of the lost HIV-negative patients had had recurrence, the incidence of this event in each group would have been 30.1/10,000 patient-months and 87.2/10,000 patient-months respectively, and the RR would have been 0.34 (95% CI = 0.16-0.74).

The distribution of the possible prognostic factors relating to recurrence in the group of lost patients, compared with those who remained in the study, is shown in Table 6.

**Table 6.** Distribution of possible prognostic factors for cervical intraepithelial neoplasia (CIN 2-3) recurrence in the group lost from the follow-up, in comparison with patients who remained in the cohort (Rio de Janeiro, Brazil, 2006)

	Lost from follow-up	Not lost from follow-up	p-value
**Age at time of performing LLETZ – mean (SD)**	31.87 (7.2)	32.5 (7.7)	0.400*
**Age at end of follow-up period – mean (SD)**	34.7 (7.4)	38.5 (8.4)	<0.0001*
**Length of follow-up in months – mean (SD)**	35.2 (20.9)	71.9 (27.5)	<0.0001†
**CIN grade treated – n (%)**			
CIN 2 (% in row; % in column)	57 (50.9-47.9)	55 (49.1-37.2)	0.072‡
CIN 3 (% in row; % in column)	60 (39.7-50.4)	91 (60.3-61.5)	
CIN 2-3§ (% in row; % in column)	2 (50.0-1.7)	2 (50.0-1.4)	
**LLETZ specimen margin involvement**			
Any involvement (% in row; % in column)	25 (39.1-21.0)	39 (60.9-26.4)	
No involvement|| (% in row; % in column)	94 (46.3-79.0)	109 (53.7-73.6))	0.309‡

SD = standard deviation; LLETZ = large loop excision of the transformation zone; CIN = cervical intraepithelial neoplasia.

* Student's t test, without assuming equal variance;

† Student's t test, assuming equal variance;

‡ Chi-squared test;

§ Not possible to differentiate between CIN-2 or 3 and therefore excluded from the statistical test of association of CIN grade with being lost to follow-up;

|| Including cases for which margin involvement could not be assessed due to thermal artifact or excessive fragmentation.

## Discussion

We found a higher risk of recurrence of CIN 2-3 in HIV-infected Brazilian women than in non-HIV-infected women. Even taking into account the wide range of the confidence interval, recurrence was at least twice as frequent as in non-HIV-infected women.

The risk of CIN 2-3 recurrence after LLETZ in our study was lower than what was observed in the cohort studied by Heard et al.^25^ They found an absolute risk of 8.6 per 100 patient-years, which is twice as high as our finding of 30.06/10,000 woman-months in HIV-infected patients (which can be converted to 3.61 per 100 patient-years). This can be partially explained by the lower median level of CD4 count.

We observed an increasing risk of recurrence over time among the HIV-positive women, which reached 26% at the 62^nd^ month. Among the HIV-negative women, this likelihood stabilized after 93 months at almost 0.6% of the women, which therefore suggests that HIV-positive patients need longer follow-ups than HIV-negative women do, and that LLETZ is an effective method for treating HIV-negative women. For HIV-positive women, the same management protocol may apply in the event of recurrence, for their retreatment.

Since CIN grade and margin involvement failed to show any significant relationship with the outcome, we did not test for confounding. Length of follow-up, however, was not equally distributed between the groups in relation to the outcome. Nonetheless, if this were a confounder, it would bias the result such that the HIV-positive group would be favored (greater length of follow-up in the HIV-negative group would show more recurrences in this group, if this factor were a confounder). CD4 count and HAART use were not statistically related to recurrence in HIV patients, but those who were using HAART seemed to have a better prognosis.

In our study groups, the only significant factor relating to recurrence of CIN 2-3 treated by LLETZ was HIV status.

We had proportionally more losses in the control group. This was due to the fact that the HIV-positive patients were followed up more closely because of their disease. Reanalysis of the censored cases showed that if all HIV-negative patients who were lost had had recurrence, our results would have been negative (RR < 1.0). However, this would not be a reasonable assumption.

The losses from the follow-up seem not to have biased our results. As shown in Table 5, the known causes of losses were not related to the outcome. The losses for which the reasons for leaving the cohort were unknown were similarly distributed between the study groups. Furthermore, the groups of lost patients and continuing patients were similar with regard to age at the time of treatment, CIN grade and margin involvement, thus showing that the losses were not related to the prognostic factors studied (Table 6).

## Conclusion

There was a higher risk of recurrence of CIN 2-3 among the HIV-infected women treated by LLETZ. HIV-positive women would need longer follow-up in order to detect and treat recurrence. LLETZ proved to be effective in treating CIN 2-3, in view of the low risk of recurrence among HIV-negative women. We found that margin status in the LLETZ specimen and CIN grade were not confounding factors. HAART use may lead to a better prognosis in relation to recurrence of CIN 2-3 among HIV patients.
